# Relationship between microplastics in stool, diet, and inflammatory markers in healthy Japanese individuals

**DOI:** 10.1265/ehpm.25-00403

**Published:** 2026-03-06

**Authors:** Mayumi Tsuji, Kazue Ishitsuka, Yasuhiro Ishihara, Chihaya Koriyama, Mieko Takahashi, Daisuke Nakajima, Yuko Mine, Tin-Tin Win-Shwe, Keisuke Shimizu, Keiko Shimizu, Sachika Akaike, Mami Kuwamura, Toshihiro Kawamoto, Kenichi Azuma

**Affiliations:** 1 Department of Environmental Health, University of Occupational and Environmental Health, Japan, 1-1 Iseigaoka, Yahatanishi-ku, Kitakyushu 807-8555, Japan; 2 Division of Life Course Epidemiology, Integrated Center for Women’s Health, National Center for Child Health and Development, 2-10-1 Okura, Setagaya-ku, Tokyo 157-8535, Japan; 3 Program of Biomedical Science, Graduate School of Integrated Sciences for Life, Hiroshima University, 1-7-1 Kagamiyama, Higashi-Hiroshima 739-8521, Japan; 4 Department of Epidemiology and Preventive Medicine, Graduate School of Medical and Dental Science, Kagoshima University, 8-35-1 Sakuragaoka, Kagoshima 890-8544, Japan; 5 Department of Administrative Nutrition, Faculty of Health and Nutrition, Tokyo Seiei College, 1-4-6 Nishishinkoiwa, Katsushika-ku, Tokyo 124-8530, Japan; 6 Health and Environmental Risk Division, National Institute for Environmental Studies (NIES), 16-2 Onogawa, Tsukuba, Ibaraki 305-8506, Japan; 7 Department of Pediatrics, School of Medicine, University of Occupational and Environmental Health, Japan, 1-1 Iseigaoka, Yahatanishi-ku, Kitakyushu 807-8555, Japan; 8 Occupational Health Research and Development Center, Japan Industrial Safety and Health Association, 5-35-2 Shiba, Minato-ku, Tokyo 108-0014, Japan; 9 Department of Preventive Medicine and Behavioral Sciences, Kindai University Faculty of Medicine, 1-14-1 Mihara-dai, Minami-ku, Sakai, Osaka 590-0197, Japan

**Keywords:** Stool microplastics, Healthy Japanese, Food intake, Oxidative stress markers, Inflammatory markers, Thymic stromal lymphopoietin, Plastic packaging, Gastrointestinal tract

## Abstract

**Background:**

Exposure to microplastics (MPs) can have adverse gastrointestinal effects by inducing inflammation and oxidative stress. The types of MPs in stool vary with the dietary intake. However, how MPs in the intestinal tract influence the inflammatory cytokine levels in the gastrointestinal tract in healthy individuals remains unclear, particularly in Japan, characterized by a high intake of vegetables and seafood. In this study, we investigated the relationship between food intake, stool MPs, and inflammatory markers in healthy Japanese individuals, and estimated the sources of the stool MPs.

**Methods:**

Twenty-two participants completed a questionnaire on daily food intake for 7 days. Thereafter, stool samples were collected to examine MP density via Fourier-transform infrared spectrophotometry. On day 8, blood samples were collected and analyzed for serum oxidative stress markers and cytokine levels. Next, the effect of total stool MP particle density (Low vs. High) on oxidative stress markers and cytokines levels was analyzed.

**Results:**

The median total MP particle density of the participants (median age: 44 years) was 7.20 MP particles per g of stool. Seafood intake was higher in the High MP group than in the Low MP group, with a Mann-Whitney U test yielding *p* = 0.035 for seafood intake. However, after the false discovery rate (FDR) correction, this effect was not significant. Nevertheless, the effect size for seafood intake was large, suggesting an association with MP level. Relative to the Low MP group, the High MP group showed significantly higher thymic stromal lymphopoietin (TSLP) levels (odds ratio: 13.5; 95% confidence interval: 0.99–183, *p* = 0.050). The analysis further revealed that the seafood consumed by the High MP group contained significant amounts of polyethylene (PE) and polypropylene (PP), MPs commonly used in plastic packaging (PE, *p* = 0.028; PP, *p* = 0.053).

**Conclusion:**

This study showed that stool MP particle density is likely associated with seafood intake and the TSLP level, implying that excessive MP intake may adversely affect human health. Therefore, measures to reduce MP exposure are urgently required.

**Supplementary information:**

The online version contains supplementary material available at https://doi.org/10.1265/ehpm.25-00403.

## 1. Introduction

Human exposure to microplastics (MPs), small plastic particles with sizes <5 mm [1], primarily occurs via the ingestion and inhalation routes [2]. Exposure via these pathways results from MP release during the decomposition of plastic products or fabrication for industrial purposes. Thus, the MPs can become suspended in the air and eventually end up in various environmental matrices, including soil, water, and food [3, 4]. MPs have also been frequently detected in seafood, salt, vegetables, and fruits, and it is well established that the plastic materials used for storage and packaging constitute important MP sources [5].

Exposure to MPs can induce cytotoxicity and adversely affect multiple organs and organ systems, including the digestive, respiratory, nervous, reproductive, and cardiovascular systems [6]. MP exposure has also been implicated in various gastrointestinal tract complications, such as digestive tract inflammation, constipation, irritable bowel syndrome, and gut microbiota disruption [2]. In addition, ingestion of MPs induces oxidative stress in the intestinal tract [7].

Additionally, MPs have been detected in various human biological samples, such as breast milk, infant feces, semen, stool, sputum, and urine. Among these biological samples, stool has been identified as the ideal candidate for investigating the effects of MPs on the digestive tract [8]. Notably, stool MPs have been associated with intestinal inflammatory biomarkers in pregnant women [9]. Therefore, measuring the levels of MPs in stool not only provides direct evidence of human exposure [10] but can also help clarify the impact of MPs on gut health.

The sizes of MPs in stool samples vary considerably depending on food intake [10, 11]. Compared with other countries, Japan has a unique dietary pattern characterized by a high intake of vegetables and seafood [12]. However, there is no report on how diet influences stool MP content, and the effects of stool MPs on the expression levels of gut inflammatory markers remain unclear. Therefore, in this study, we aimed to investigate the relationship between diet, stool MPs, and gut inflammatory markers, focusing on healthy Japanese individuals. We also aimed to estimate the sources of stool MPs. The findings of this study may provide valuable insights regarding the impacts of MPs on gut health and highlight strategies to mitigate MP exposure.

## 2. Methods

### 2.1 Study participants and procedures

In 2023, 22 healthy individuals, comprising 21 university administrative staff and one hospital employee, all residing in Fukuoka, Japan, were enrolled for this study. The exclusion criteria included those on a diet regimen due to a medical condition within the last 4 weeks; occurrence of diarrhea or obstipation within the previous 2 weeks; antibiotic use within the last 2 weeks; intake of medications that influence stool frequency and consistency within the last 2 weeks; intake of medications that affect resorption within the last 2 weeks; a diagnosis of gastrointestinal disease; and alcohol abuse, defined as an Alcohol Use Disorders Identification Test (AUDIT) score >10; and age >65 years [13]. Among the 22 included participants, 17 participated in the study in October 2023, while the remaining five participated in November 2023. Following a previously reported procedure, the participants were provided with a stool sampling kit and asked to complete a food intake questionnaire daily for 7 days before stool and blood samples were collected on days 7 and 8, respectively. No dietary restrictions were imposed [13, 14].

### 2.2 Dietary assessment

Participants were allowed to eat and drink without restrictions. Food intake was monitored over 7 consecutive days using a photographic dietary recording method.

**Fig. 1:**
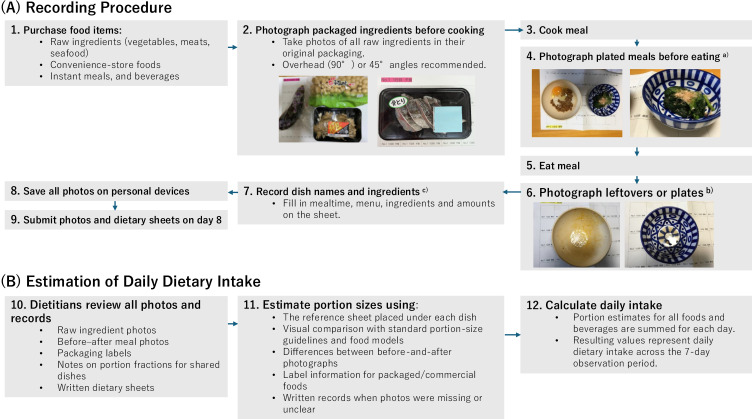
Detailed procedures and estimation process of daily food intake a) ◦ Place the dish on the designated reference sheet. ◦ Take photos from multiple angles: overhead, 45°, and sideways (to capture depth). b) ◦ Overhead and 45° views. ◦ If no leftovers remain, only an overhead photo is required. ◦ For beverages, photograph the remaining amount from the side. c) ◦ Use the paper-based dietary sheet. ◦ Note all identifiable ingredients; use the reverse side if needed. ◦ For shared dishes, record the estimated fraction consumed (e.g., “¼ of stir-fried vegetables”). ◦ For commercial foods, photograph the product name, brand, and ingredient labels. ◦ For meals outside the home, photograph the restaurant name and menu items.

Figure 1 shows the detailed procedures and estimation process. At each eating occasion, participants photographed all ingredients before cooking and all prepared meals before and after eating, and documented dish names and identifiable ingredients on a paper-based dietary sheet [15, 16]. All photographs were stored on personal devices and collected on day 8 together with the written records.

Trained dietitians estimated portion sizes and calculated daily food intake by reviewing the submitted photographs and dietary record sheets. Based on the Standard Tables of Food Composition in Japan, we estimated the daily intake for each of the 29 food groups [17].

### 2.3 Stool sample collection and assessment of stool MP content

Stool sampling was performed in accordance with a predefined protocol to avoid sample contamination with plastics or synthetic fibers [13, 14]. The participants defecated on a water-soluble sheet of paper, and using a metal spoon, transferred fresh stool, not mixed with urine (approximately 25 g), into labelled, pseudonymized pre-weighed glass bottles containing an aqueous antibacterial solution [13, 14]. Thereafter, the glass bottles were immediately covered and shipped to IDEA Consultants, Inc., Institute of Environmental Ecology for the quantification of MPs particles as previously described [13, 14]. In brief, the samples were shaken at 150 rpm for 12 h in a reciprocating shaker for homogenization. Thereafter, 3 g of each sample was subjected to a Fenton-based oxidation treatment for 7 days, followed by alkali treatment with 0.05 M NaOH for 3 days. Next, indigestible materials and residual MPs were collected by filtration through a 10-µm stainless-steel filter and examined using Fourier transform infrared spectrophotometry (FT-IR) using the IRAffinity-1S/AIM-9000 system (Shimadzu, Kyoto, Japan). Notably, MPs with a size >20 µm were examined. Data from five blanks, used during MPs measurements, showed the presence of PE (0.7–1.7 particles in three blanks), PP (0.3–3 particles in four blanks), PMMA (0.7 particles in one blank), PU (0.3 particles in one blank), and PVC (0.3 particles in two blanks). The mean counts of PE, PP, PMMA, PU, and PVC detected in the blanks (0.62, 0.86, 0.14, 0.06, and 0.12 particles, respectively) were subtracted from each sample. After blank correction, negative values were set to zero.

### 2.4 Blood sample collection and measurement of oxidative stress markers and inflammatory cytokines in serum

Blood samples were collected, allowed to clot for approximately 1 h, and thereafter centrifuged at 3,000 × g for 15 min. The resulting serum samples were then transferred to clean tubes and stored at −80 °C until further analysis. 8-hydroxy-2′-deoxyguanosine (8-OHdG) and oxidized low-density lipoprotein (ox-LDL) in the serum were measured using a highly sensitive ELISA kit for 8-OHdG (The Japan Institute for the Control of Aging, Shizuoka, Japan) or Human ox-LDL ELISA kit (The Japan Institute for the Control of Aging), respectively, according to the manufacturers’ instructions. To evaluate the inflammatory cytokines, the serum levels of inflammatory cytokines, namely, interleukin (IL)-1α, IL-1β, IL-11, IL-12p40, IL-12p70, IL-15, IL-18, IL-23, IL-27, IL-33, granulocyte-macrophage colony-stimulating factor (GM-CSF), thymic stromal lymphopoietin (TSLP), and interferon (IFN)-α2 were quantified using LEGENDplex™ Human Cytokine Panel 2 (BioLegend, San Diego, CA, USA) in conjunction with a CytoFLEX S flow cytometer (Beckman-Coulter, Tokyo, Japan), as previously described [18].

### 2.5 Statistical analyses

All statistical analyses were performed using Stata software v15 (Stata Corp., College Station, TX, USA). For the two-group comparison, the Mann-Whitney U test and multivariable logistic regression analysis were performed. To account for multiple testing, p values obtained from the Mann–Whitney U test were adjusted using the false discovery rate (FDR) procedure. Effect sizes were calculated using Cliff’s delta (δ), where values close to 0 indicate substantial overlap between groups, whereas values closer to ±1 indicate stronger dominance. Effect sizes were interpreted as negligible (|δ| < 0.147), small (0.147–0.33), medium (0.33–0.474), or large (≥0.474), according to previously proposed guidelines [19, 20]. Statistical significance (two-sided) was set at *p* < 0.05.

Oxidative stress markers and cytokine levels below the detection limit were attributed to the level at half the detection limit value. *p*-values obtained following the multivariable logistic regression analysis were adjusted for age, sex, and drinking habit, which are not only basic moderators but also associated with inflammation [21–25]. Further, before statistical analysis, the participants were grouped based on total MP particle density (Low and High MP groups) and oxidative stress markers and cytokine levels (Low and High biomarker groups). We divided all subjects into two groups based on total MP particle density and biomarker levels automatically, so that each sub-group would consist of approximately 1/2 of the total subjects. When the median value was shared by multiple participants (e.g., in a cohort of 22 participants), group sizes were allowed to differ slightly (e.g., 12 vs. 10 participants).

## 3. Results

### 3.1 Participant characteristics

The median age of the 22 participants was 44 years (males, 39 years; females, 51 years), and their food intake, including green tea, black tea, coffee, and white rice, was >100 g per day. Further, females consumed significantly higher amounts of animal fats and oils, brown rice, fruits, beverages (including green tea, black tea, coffee), and nuts than males (*p* = 0.025, 0.042, 0.032, 0.021, and 0.021, respectively) (Table 1).

**Table 1:** Characteristics of the study population

	**Number (%) /Median (25th and 75th percentiles)**		

	**Total (n = 22)**	**Male (n = 10)**	**Female (n = 12)**
**Age (year)**	44 (36, 52)	39 (28, 44)	51 (43, 59)
**Smoking**			
Never smoker or Ex-smoker	22 (100%)	10 (100%)	12 (100%)
Current smoker	0 (0%)	0 (0%)	0 (0%)
**Drinking habits**			
No	12 (55%)	4 (40%)	8 (67%)
Yes	10 (45%)	6 (60%)	4 (33%)
**Drink quantity (mL/day)**	500 (88, 660)	500 (175, 1000)	200 (30, 575)
**Dietary components (g/day)**			
Alcoholic drinks	0.5 (0.0, 18.3)	0.7 (0.0, 43.2)	0.3 (0.0, 0.9)
Animal fats and oils	1.1 (0.0, 1.7)	0.1 (0.0, 1.0)	1.5 (1.0, 2.0)
Beans	40.5 (10.5, 65.5)	43.1 (15.2, 118.6)	37.9 (8.2, 57.2)
Breads	9.2 (0.0, 47.4)	0.0 (0.0, 24.3)	16.9 (0.0, 59.6)
Brown rice	0.0 (0.0, 0.3)	0.0 (0.0, 0.0)	0.1 (0.0, 1.4)
Carbonated drinks, fermented milk drink	0.0 (0.0, 47.5)	0.0 (0.0, 132.9)	0.0 (0.0, 35.4)
Confectionaries	35.8 (23.7, 67.1)	33.2 (21.3, 69.5)	39.1 (28.9, 70.7)
Dairy products	46.5 (16.0, 147.9)	20.4 (11.1, 62.4)	109.2 (24.7, 246.6)
Eggs	19.5 (8.8, 33.5)	13.2 (7.5, 25.2)	22.9 (12.3, 45.7)
Fruit juice, vegetable juice	0.0 (0.0, 19.4)	0.0 (0.0, 0.0)	0.0 (0.0, 61.2)
Fruits	5.6 (0.8, 60.9)	2.3 (0.2, 4.9)	29.6 (2.7, 91.5)
Grains (excluding rice)	7.1 (1.9, 12.7)	8.4 (1.8, 19.5)	5.5 (1.8, 12.3)
Green and yellow vegetables	57.3 (18.9, 100.6)	30.3 (10.0, 94.4)	71.6 (49.1, 105.4)
Green tea, black tea, coffee	614.6 (422.5, 818.3)	458.3 (191.0, 653.3)	772.3 (576.8, 949.6)
Light-colored vegetables	65.4 (46.4, 120.8)	61.5 (39.5, 87.7)	79.8 (49.6, 123.9)
Meat	98.4 (55.7, 114.4)	105.6 (76.2, 112.4)	68.3 (34.1, 125.4)
Mushrooms	6.1 (0.0, 10.7)	6.1 (3.2, 10.7)	5.9 (0.0, 12.6)
Noodles	64.0 (24.9, 109.6)	58.9 (12.5, 83.0)	65.5 (29.3, 134)
Nuts	0.7 (0.1, 1.5)	0.3 (0.0, 0.9)	1.2 (0.5, 2.2)
Pickles	1.6 (0.0, 4.9)	1.6 (0.0, 4.1)	2.2 (0.1, 5.9)
Potatoes	16.4 (1.4, 44.5)	21.7 (0.2, 44.5)	12.9 (1.8, 47.8)
Processed foods	0.0 (1.3, 30.8)	0.0 (1.3, 35.8)	0.0 (1.4, 23.8)
Seafood	29.9 (19.3, 46.0)	29.6 (16.0, 48.8)	29.9 (18.2, 47.1)
Seasoning	71.3 (51.3, 117.0)	101.9 (51.3, 123.3)	66.9 (36.4, 87.5)
Seaweed	1.0 (0.1, 3.7)	2.0 (0.1, 3.7)	0.9 (0.1, 3.7)
Sugar, jams, sweeteners	1.6 (0.8, 3.7)	1.3 (0.8, 3.0)	1.8 (0.7, 5.2)
Vegetable oils	13.3 (8.8, 18.6)	14.6 (7.8, 19.4)	11.8 (9.0, 18.0)
White rice	230.8 (144.5, 302.5)	254.0 (234.6, 325.4)	156.7 (136.4, 275.2)

Data are presented as number (%) / median (25%, 75%).

### 3.2 Particle density of MPs in stool

All the participants had MPs in their stools. Specifically, 15 MP types were detected: including ethylene-acrylic acid copolymer (EAA), ethylene-ethyl acrylate copolymer (EEA), ethylene-vinyl acetate copolymer (EVA), ionomer (IO), polyethylene (PE), polyethylene oxide (PEO), polyethylene terephthalate (PET), poly methyl methacrylate (PMMA), polymethyl pentene (PMP), polypropylene (PP), polystyrene (PS), polyurethane (PU), polyvinyl chloride (PVC), poly vinyl stearate (PVS), and silicone (SI). The median total MP particle density was 7.20 particles per gram of stool (25th to 75th percentile: 3.05–15.63). Further, no statistically significant differences were observed between males and females in terms of stool MP particle density (males: median = 5.55; 25th to 75th percentile, 2.53–15.63 vs. females: median = 9.25; 25th to 75th percentile, 3.25–22.70; *p* = 0.391). Details in this regard are provided in Table 2.

**Table 2:** Density of microplastic particles in stool samples (per gram of stool)

			**Microplastic particle density (per gram of stool)**																			

**ID**	**Sex**	**Total stool** **weight (g)**	**EAA**	**EEA**	**EP**	**EVA**	**EVOH**	**IO**	**PA**	**PE**	**PEO**	**PET**	**PMMA**	**PMP**	**PP**	**PS**	**PTFE**	**PU**	**PVC**	**PVS**	**SI**	**Total**
1	F	12.17	-	-	-	0.66	-	0.66	-	4.70	-	-	-	-	3.13	-	-	-	-	-	-	9.15
2	F	24.49	-	-	-	-	-	-	-	5.99	-	-	-	-	9.05	-	-	-	-	-	-	15.04
3	M	21.83	-	-	-	1.66	-	-	-	4.37	-	-	-	-	9.13	-	-	-	-	-	-	15.16
4	M	17.90	-	-	-	-	-	-	-	0.05	-	-	0.19	-	5.14	-	-	-	-	-	-	5.38
5	M	8.57	-	-	-	-	-	-	-	0.37	-	-	0.19	-	2.12	-	-	-	-	-	-	2.68
6	F	25.02	-	-	-	-	-	-	-	12.72	-	-	-	-	2.47	-	-	-	-	-	-	15.19
7	M	19.42	-	-	-	-	-	-	-	5.72	-	0.33	-	-	1.14	-	-	-	-	-	-	7.19
8	F	12.52	-	-	-	-	-	-	-	-	-	-	-	-	44.10	-	-	-	-	-	-	44.10
9	F	8.37	1.67	1.67	-	3.33	-	-	-	7.71	-	-	1.53	1.67	12.47	-	-	-	-	-	-	30.05
10	F	19.93	-	3.67	-	-	-	-	-	1.38	-	-	0.53	-	2.14	-	-	0.24	-	1.33	-	9.29
11	F	16.24	-	0.33	-	0.33	-	-	-	1.70	0.33	-	-	-	-	-	-	-	-	0.33	0.33	3.35
12	M	25.12	-	-	-	-	-	-	-	1.38	-	0.33	-	-	-	-	-	-	-	-	0.33	2.04
13	F	10.74	0.67	-	-	0.67	-	-	-	3.38	-	-	-	-	1.81	-	-	-	-	-	0.67	7.20
14	M	11.74	-	-	-	1.67	-	-	-	2.73	-	-	-	-	12.52	-	-	-	-	-	-	16.92
15	F	7.65	-	-	-	0.33	-	0.33	-	1.05	-	-	-	0.67	0.81	-	-	-	-	-	-	3.19
16	F	19.09	-	-	-	-	-	-	-	9.38	-	-	-	-	14.14	-	-	-	-	-	1.67	25.19
17	M	13.62	-	23.23	-	-	-	-	-	20.95	-	1.66	-	-	-	-	-	-	-	-	-	45.84
18	M	10.61	-	0.67	-	-	-	-	-	0.05	-	-	-	-	-	-	-	-	-	0.67	-	1.39
19	M	18.35	0.33	-	-	-	-	-	-	4.66	-	0.33	-	-	-	-	-	-	-	0.33	-	5.65
20	M	21.79	0.33	-	-	0.33	-	0.33	-	1.67	-	-	-	-	0.12	0.33	-	-	-	-	-	3.11
21	F	11.80	-	-	-	0.33	-	-	-	0.05	-	-	-	-	-	-	-	-	0.21	-	-	0.59
22	F	27.18	-	-	-	-	-	0.33	-	1.05	-	-	-	-	1.47	-	-	-	-	-	-	2.85

M, male; F, female; EAA, ethylene-acrylic acid copolymer; EEA, ethylene-ethyl acrylate copolymer; EP, epoxy resin; EVA, ethylene-vinyl acetate copolymer; EVOH, ethylene-vinyl alcohol copolymer; IO, ionomer; PA, polyamide; PE, polyethylene; PEO, polyethylene oxide; PET, polyethylene terephthalate; PS, polystyrene; PTFE, polytetrafluoroethylene; PMMA, polymethyl methacrylate; PMP, polymethylpentene; PP, polypropylene; PU, polyurethane; PVC, polyvinyl chloride; PVS, polyvinyl stearate; SI, silicone.

**Fig. 2:**
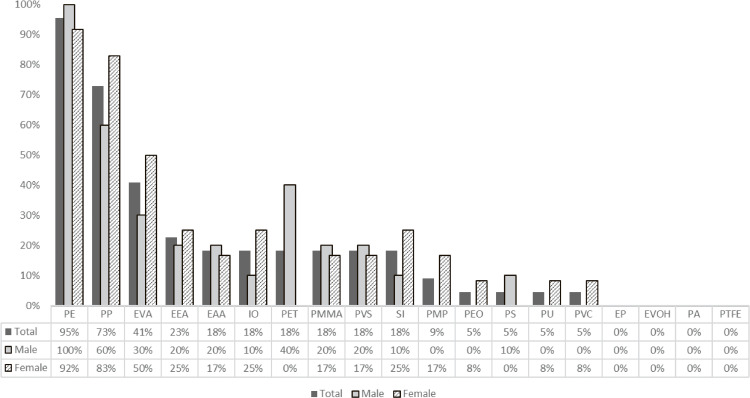
Frequency of the occurrence of 19 microplastic types in stool samples from 22 healthy Japanese

For all 22 participants, PE was the most frequently observed MP in the analyzed samples (95%), followed by PP (73%) and EVA (41%). Additionally, among all the MPs with overall detection frequency ≥10%, PET was only detected in males, whereas SI was detected at a higher rate in females than in males (Fig. 2).

### 3.3 Food intake amount and stool MP particle density

As shown in Table 3, seafood intake was higher in the High MP group than in the Low MP group (High MP group: median = 43.0 g/day; 25th to 75th percentile, 30.1–60.9 vs. Low MP group: median = 21.7 g/day; 25th to 75th percentile, 17.6–33.0). Mann-Whitney U tests were performed for each food item, with seafood intake showing a significant association (*p* = 0.035). After FDR correction, the association was no longer statistically significant, although the effect size for seafood intake was large (Cliff’s delta = 0.533).

**Table 3:** Relationship between dietary intake and stool microplastic particle density

	**Number of microplastic particles** **(per gram of stool)**				

**Food intake amount (g/day)** **Median (25th, 75th)**	**Low MP group** **(N = 12)**	**High MP group** **(N = 10)**	*p* value*	***FDR-adjusted*** p value**	**Effect size** **(Cliff’s delta)^†^**
Alcoholic drinks	0.3 (0, 11.1)	0.5 (0, 70.0)	0.706	0.927	0.092
Animal fat oils	0.6 (0, 1.7)	1.3 (0, 2.2)	0.402	0.927	0.217
Beans	37.9 (6.1, 155.3)	44.3 (13.4, 61.2)	0.792	0.927	0.067
Breads	4.2 (0, 52.0)	10.4 (0, 47.4)	0.890	0.927	0.033
Brown rice	0.0 (0.0, 0.3)	0.0 (0.0, 0.9)	0.811	0.927	0.050
Carbonated drinks, fermented milk drink	0 (0, 89.7)	0 (0, 47.5)	0.878	0.927	0.033
Confectionaries	37.1 (21.1, 78.4)	35.8 (26.8, 65.0)	0.895	0.927	0.033
Dairy products	39.6 (20.1, 233.4)	65.3 (8.0, 135.6)	0.598	0.927	−0.133
Eggs	22.8 (8.9, 45.7)	13.7 (8.6, 25.6)	0.235	0.815	−0.300
Fruit juice, vegetable juice	0 (0, 41.3)	0 (0, 19.4)	0.967	0.967	−0.001
Fruits	13.5 (0.8, 75.3)	2.8 (0.8, 16.9)	0.448	0.927	−0.192
Grains, excluding rice	4.8 (1.4, 11.0)	8.0 (2.2, 13.3)	0.429	0.927	0.200
Green and yellow vegetables	58.5 (16.0, 98.1)	55.7 (32.3, 101.4)	0.869	0.927	0.042
Green tea, black tea, coffee	458.3 (255.5, 782.4)	741.2 (598.4, 953.3)	0.056	0.686	0.483
Light-colored vegetables	60.5 (40.5, 118.2)	74.7 (53.5, 120.8)	0.598	0.927	0.133
Meat	85.8 (30.0, 110.9)	108.0 (67.4, 127.9)	0.187	0.775	0.333
Mushrooms	4.6 (0, 8.1)	8.2 (4.0, 14.1)	0.134	0.686	0.375
Noodles	52.7 (15.1, 116.1)	64.0 (37.1, 88.4)	0.644	0.927	0.117
Nuts	0.7 (0.2, 1.6)	0.7 (0.1, 1.5)	0.791	0.927	−0.050
Pickles	1.4 (0, 3.9)	2.5 (0.2, 6.1)	0.369	0.927	0.225
Potatoes	11.5 (0.1, 26.6)	24.5 (4.2, 56.0)	0.113	0.686	0.400
Processed foods	1.3 (0, 33.7)	1.4 (0, 30.8)	0.833	0.927	−0.050
**Seafood**	**21.7 (17.6, 33.0)**	**43.0 (30.1, 60.9)**	**0.035**	**0.686**	**0.533**
Seasoning	56.3 (31.9, 117.7)	84.9 (66.1, 120.7)	0.253	0.815	0.300
Seaweed	0.7 (0, 3.9)	1.7 (0.2, 3.4)	0.487	0.927	0.175
Sugar, jams, sweeteners	1.4 (0.7, 3.6)	1.9 (0.8, 4.0)	0.575	0.927	0.150
Vegetable oils	10.9 (5.2, 15.9)	14.9 (11.5, 19.4)	0.106	0.686	0.417
White rice	230.8 (137.9, 302.5)	223.7 (151.0, 304.2)	0.843	0.927	0.050
Drink quantity (mL/day)	500 (100, 960)	250 (30, 500)	0.142	0.686	−0.367

*: *p* values obtained based on the Mann-Whitney U test.

**: *p* values were adjusted using the false discovery rate (FDR).

^†^: Cliff’s delta represents the effect size of the Mann–Whitney U test; positive values indicate higher intake in the high MP group.

**Fig. 3:**
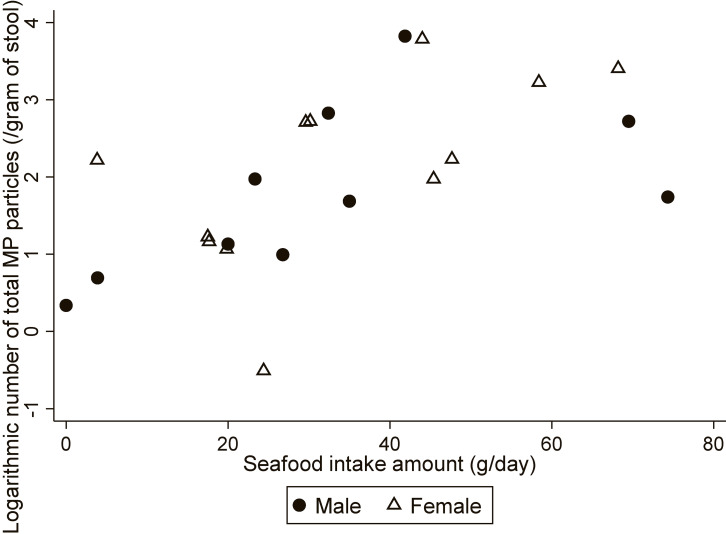
Relationship between daily seafood intake and number of total microplastic particles

Because the number of total MP particles was skewed, the variables were treated with natural logarithmic conversion before processing. We then created a scatter plot showing the number of MP particles and fish intake amount as continuous variables. Consequently, the relationship between the amount of seafood intake and number of MP particles can be seen in the scatter plot. Spearman correlation revealed a statistically significant association between seafood intake and the total number of MP particles (total: r = 0.646, *p* < 0.005; male: r = 0.709, *p* = 0.022; female: r = 0.650, *p* = 0.022) (Fig. 3). Although not statistically significant, green tea/black tea/coffee intake was higher in the High MP group than in the Low MP group (High MP group: median = 741.2 g/day; 25th to 75th percentile, 598.4–953.3 vs. Low MP group: median = 458.3 g/day; 25th to 75th percentile, 255.5–782.4; *p* = 0.056).

### 3.4 Oxidative stress markers, cytokine levels in serum, and MP particle density

The High and Low MP groups showed no significant differences in the oxidative stress markers and cytokine levels (Table 4). Then, to assess the association between oxidative stress markers, cytokine levels, and stool MP particle density, we performed multivariable logistic regression analyses. The results thus obtained (Table 5) showed that relative to the Low TSLP group, the High TSLP group showed a significantly higher stool MP particle density (odds ratio [OR]: 13.5; 95% confidence interval [CI], 0.99–183; *p* = 0.050). No significant differences were observed for all the other biomarkers.

**Table 4:** Relationship between oxidative stress markers, cytokines levels and stool microplastic particle density

		**Number of microplastic particles** **(per gram of stool)**				

**Median biomarker** **levels (pg/mL)** **median (25th, 75th)**	**Total (N = 22)**	**Low MP group** **(N = 12)**	**High MP group** **(N = 10)**	*p* value*	***FDR-adjusted*** p value**	**Effect size** **(Cliff’s delta)^†^**
8OH-dG	0.18 (0.17, 0.20)	0.17 (0.16, 0.20)	0.19 (0.17, 0.27)	0.103	0.386	0.408
ox-LDL	34.0 (28.3, 70.0)	38.4 (29.8, 89.4)	30.8 (27.1, 60.1)	0.262	0.786	−0.283
IFN-α2	5.08 (1.71, 27.7)	5.01 (1.29, 22.3)	12.1 (1.71, 36.6)	0.429	0.804	0.200
TSLP	0.38 (0.24, 0.90)	0.24 (0.24, 0.53)	0.77 (0.24, 1.79)	0.057	0.386	0.450
IL-1α	1.30 (0.67, 3.14)	1.29 (0.72. 2.38)	1.56 (0.66, 3.37)	0.717	0.895	0.092
IL-1β	1.66 (0.81, 3.01)	1.49 (0.47, 2.72)	1.66 (0.86, 4.10)	0.620	0.895	0.125
GM-CSF	0.52 (0.52, 1.32)	0.52 (0.52, 1.03)	1.21 (0.52, 1.70)	0.089	0.386	0.383
IL-11	7.80 (2.36, 11.5)	5.00 (2.36, 10.5)	8.84 (2.36, 17.7)	0.406	0.804	0.200
IL-12p40	572 (233, 1469)	572 (236, 1442)	817 (218, 1507)	0.895	0.895	0.033
IL-12p70	1.32 (0.90, 2.10)	1.61 (0.95, 2.19)	1.26 (0.80, 1.56)	0.373	0.804	−0.225
IL-15	127 (56.6, 253)	127 (63.3, 235)	164 (45.5, 264)	0.843	0.895	0.050
IL-18	76.8 (61.5, 106)	62.7 (60.9, 115)	78.7 (62.8, 103)	0.692	0.895	0.100
IL-23	5.54 (2.64, 7.95)	7.47 (5.00, 7.96)	2.97 (1.97, 7.15)	0.065	0.386	−0.467
IL-27	58.2 (30.4, 200)	58.2 (37.2, 166)	102 (26.4, 289)	0.792	0.895	0.067
IL-33	21.5 (7.78, 51.5)	18.4 (7.28, 40.4)	29.3 (6.90, 65.5)	0.644	0.895	0.117

** *p* values obtained based on the Mann-Whitney U test.

**: p values were adjusted using the false discovery rate (FDR).

^†^: Cliff’s delta represents the effect size of the Mann–Whitney U test; positive values indicate higher intake in the high MP group.

**Table 5:** Effect of total stool microplastic particle density on oxidative stress markers and cytokine levels

	**oxidative stress** **markers,** **cytokines levels**		**Crude model**		**Adjusted model**		**oxidative stress** **markers,** **cytokines levels**		**Crude model**		**Adjusted model**		**oxidative stress** **markers,** **cytokines levels**		**Crude model**		**Adjusted model**	
	**Low**	**High**	**OR ** **(95% CI)**	**p value**	**OR ** **(95% CI)**	p value*	**Low**	**High**	**OR ** **(95% CI)**	**p value**	**OR ** **(95% CI)**	p value*	**Low**	**High**	**OR ** **(95% CI)**	**p value**	**OR ** **(95% CI)**	p value*
**Number of microplastic** **particles (per gram of stool)**	**8OH-dG**						**ox-LDL**						**IFN-α2**					
		
**Low MP group**	8	4	1.00 (reference)		1.00 (reference)		4	8	1.00 (reference)		1.00 (reference)		6	6	1.00 (reference)		1.00 (reference)	
**High MP group**	5	5	1.76 (0.36, 11.2)	0.431	5.12 (0.41, 63.4)	0.204	7	3	0.21 (0.04, 1.31)	0.095	0.13 (0.01, 1.53)	0.105	5	5	1.00 (0.19, 5.36)	1.000	6.65 (0.23, 190)	0.268

	**TSLP**						**IL-1α**						**IL-1β**					
		
**Low MP group**	8	4	1.00 (reference)		1.00 (reference)		6	6	1.00 (reference)		1.00 (reference)		6	6	1.00 (reference)		1.00 (reference)	
**High MP group**	3	7	4.67 (0.77, 28.5)	0.095	13.5 (0.99, 183)	0.050	5	5	1.00 (0.19, 5.36)	1.000	1.50 (0.17, 13.1)	0.717	5	5	1.00 (0.19, 5.36)	1.000	2.51 (0.24, 26.6)	0.444

	**GM-CSF**						**IL-11**						**IL-12p40**					
		
**Low MP group**	9	3	1.00 (reference)		1.00 (reference)		7	5	1.00 (reference)		1.00 (reference)		6	6	1.00 (reference)		1.00 (reference)	
**High MP group**	4	6	4.50 (0.73, 27.7)	0.105	12.7 (0.81, 198)	0.070	4	6	2.10 (0.38, 11.6)	0.395	3.24 (0.41, 25.3)	0.262	5	5	1.00 (0.19, 5.36)	1.000	6.65 (0.23, 190)	0.268

	**IL-12p70**						**IL-15**						**IL-18**					
		
**Low MP group**	6	6	1.00 (reference)		1.00 (reference)		6	6	1.00 (reference)		1.00 (reference)		7	5	1.00 (reference)		1.00 (reference)	
**High MP group**	5	5	1.00 (0.19, 5.36)	1.000	1.06 (0.16, 6.84)	0.954	5	5	1.00 (0.19, 5.36)	1.000	6.65 (0.23, 190)	0.268	4	6	2.10 (0.38, 11.6)	0.395	3.93 (0.36, 43.4)	0.264

	**IL-23**						**IL-27**						**IL-33**					
		
**Low MP group**	4	8	1.00 (reference)		1.00 (reference)		6	6	1.00 (reference)		1.00 (reference)		7	5	1.00 (reference)		1.00 (reference)	
**High MP group**	7	3	0.21 (0.04, 1.31)	0.095	0.17 (0.02, 1.44)	0.105	5	5	1.00 (0.19, 5.36)	1.000	2.73 (0.25, 29.5)	0.407	4	6	2.10 (0.38, 11.6)	0.395	3.24 (0.41, 25.3)	0.262

* p values were obtained by multivariable logistic regression analyses adjusted for age, sex and drinking habits.

To visualize a potential dose–response relationship, TSLP levels, which showed a highly skewed distribution with clustering at low values, were categorized into tertiles and plotted against the total number of MP particles (Supplementary Fig. 1). The median (25th–75th percentile) values were 3.40 (2.00–9.20) in the low tertile group (n = 11), 7.50 (4.45–27.55) in the middle tertile group (n = 4), and 15.20 (7.20–16.90) in the high tertile group (n = 7). Differences among the three groups were evaluated using the Kruskal–Wallis test, which showed no statistically significant difference (P = 0.154).

## 4. Discussion

This study had two key findings. First, MP particle density showed a positive relationship with seafood intake, consistent with previously reported data [26, 27]. Second, stool MP particle density may affect TSLP levels.

Reportedly, human exposure to MPs can result from the ingestion of seafood contaminated with MPs owing to marine plastic pollution [28], which is most severe in Asian seas [28]. Further, seafood in which MPs have been previously detected include mollusks, crustaceans, and fish [2]. In our study, the median fish intake of male and female participants was 21.7 and 43 g of fish per day, respectively. These intake amounts are considerably lower than the average amounts for men and women in Japan (137 and 108 g per day, respectively) [29], suggesting that the contribution of fish-derived MPs to the MP contents of the analyzed stools for our cohort may be lower than the average for the Japanese population.

Although we suggested a positive relationship between seafood intake and stool MP particle density, it remains unclear whether the MPs in the stool samples only originated from seafood. MPs have been detected in the skin, gills, stomach, liver, and intestines of fish [30, 31], and are therefore likely to be absent in edible fillets or seafood flesh [30]. Until present, the relationship between food intake patterns and stool MP type remains debatable, and the use of plastics in food packaging and preparation is significantly associated with stool MP content [11, 26]. Additionally, in recent years, PE and PP, as representative plastics, have been extensively used as food packaging materials [32, 33]. Their use in seafood packaging is also common [34, 35], owing to their advantages such as their ability to maintain freshness, extend shelf life, and prevent contamination [36, 37]. In this study, PE and PP were detected in over 70% of subjects, and further grouping according to individual stool PE or PP contents, i.e., the Low PE and PP detection groups or and High PE or PP detection groups, showed a significant correlation between stool PE content and seafood intake (High PE group: median = 41.9 g/day, 25th to 75th percentile, 29.6–68.2 vs. Low PE group: median = 20.7 g/day; 25th to 75th percentile, 17.5–35.0, *p* = 0.028) (Supplementary Table 1). Additionally, the High PP group showed a non-statistically significant trend toward a higher seafood intake than the Low PP group (High PP group: median = 35.0 g/day; 25th to 75th percentile, 29.6–58.4 vs. Low PP group: median = 20.0 g/day; 25th to 75th percentile, 17.5–41.9; *p* = 0.053) (Supplementary Table 1). Therefore, the significant relationship between stool MP particle density and seafood intake may be due to the attachment of MPs from the plastic containers used in their packaging to the seafood. Therefore, reducing the use of plastic packaging or going plastic-free are plausible strategies to reduce MP exposure. However, implementing such measures requires the availability of alternatives as well as a fundamental shift in existing business models [38]. Widespread adoption in the industrial sector is also yet to be achieved [38].

TSLP, a cytokine produced by intestinal epithelial cells, has been shown to exert protective effects in dextran sulfate sodium (DSS)-induced colitis models. Notably, TSLP administration via recombinant lactic acid bacteria delays disease activity, reduces histological damage, and decreases the production of pro-inflammatory cytokines, such as IFN-γ [39]. Additionally, in patients with ulcerative colitis, lower TSLP expression levels are associated with increased disease severity, suggesting that TSLP plays a protective role by promoting Treg responses and maintaining intestinal tolerance [40]. In contrast, TSLP has been implicated in accelerated tumor progression in colorectal cancer, which is often associated with colitis, and reportedly, TSLP-deficient mice show reduced tumor numbers and sizes, suggesting that this cytokine possibly contributes to tumor progression by activating intestinal epithelial cells [41]. Therefore, the role of TSLP in the intestine, which may depend on the intestinal environment, remains controversial. In this study, serum TSLP levels were higher in the High-MP group than in the Low-MP group, and although there was no evidence that the MPs regulate TSLP transcription, the TSLP promoter contains several functional binding sites for transcription factors, such as NF-κB, activator protein 1 (AP1), STAT, and Smad, which function in intestinal epithelial cells [42]. Reportedly, exposing mice to PS-MPs induces oxidative stress and inflammation and activates the NF-κB/NLRP3/IL-1β/MLCK pathway, which contributes to intestinal barrier dysfunction [7]. Although animal experiments examining the degree of inflammation in the large intestine have not clarified whether there are gender differences, sex differences have been reported in intestinal barrier function and immune responses [43, 44]. Therefore, it is possible that MPs activate TSLP, which subsequently influences several gut pathophysiological pathways, and the extent of its influence may differ between the sexes. Importantly, several types of MPs can be absorbed via the intestinal tract. Regardless, owing to the protective role of intestinal mucus and the ability of the intestinal tract to excrete a considerable proportion of ingested MPs, not all ingested particles result in systemic exposure [45, 46]. Regardless, further studies on the pharmacokinetics of MPs ingested via food intake are necessary to clarify their health effects. It is also important to conduct sex-specific animal and epidemiological studies to explore the relationship between ingested MPs and their effects on the intestinal tract via TSLP pathways. ox-LDL is often used as an indicator of oxidative stress, but while increases in ox-LDL are clear in diseased groups, there is debate concerning its reliability in assessing oxidative stress in healthy individuals [47]. In this study, we used 8-OHdG in addition to ox-LDL. Since no statistically significant differences were observed in ox-LDL or 8-OHdG, we concluded that there was no correlation between MP exposure and oxidative stress.

The High MP group showed a higher green tea/black tea/coffee intake than the low-MP group. Previous studies have shown that tea brewing releases a significant number of MPs from plastic tea bags [48, 49]. In this study, we did not investigate whether the participants used tea bags or filters to brew green tea, black tea, or coffee. However, given the increased prevalence of tea bags in recent years [50], it is highly probable that the observed stool MPs originated from tea bags. Reportedly, pre-washing is effective for reducing MP exposure [51]. Therefore, choosing loose-leaf tea or prewashing tea may be effective for reducing MP exposure to MPs.

This study had some limitations. First, we used traditional FT-IR spectroscopy, which is disadvantageous owing to its limited efficacy in the detection of small-sized MPs. To detect MPs with sizes <20 µm, different approaches, such as laser direct infrared spectroscopy (LD-IR) and thermal desorption-gas chromatography-mass spectrometry with a tubular furnace (TD-GC-MS), are required [26]. Second, generally, the MPs in seafood have sizes ≥20 µm, meanwhile those with sizes <10 µm have been detected in *Mytilus edulis*, which is commonly consumed in the US and Europe [52]. Because the MP content in stool may vary depending on the type of seafood consumed regardless of its amount, future studies should also focus on the type of seafood consumed. Third, the influence of environmental MPs could not be eliminated. Due to the ubiquitous presence of MPs in the environment, it is difficult to separate environmental MPs from sample contamination [53]. Future studies will need to address environmental MP contamination during sample processing to generate more reproducible and reliable data. Fourth, we did not obtain data regarding MP exposure via the inhalation route. Previous studies have suggested that inhalable microplastics, especially those 1–10 µm in size, can be deposited in the lungs and transferred to the bloodstream [54]. Therefore, in addition to oral ingestion, inhalation exposure may contribute to the MP burden in the body and potentially affect human health. Fifth, in this study, stools were collected on day 7. However, the correlation between MP in stool and seafood intake may change depending on the day of stool collection. Sixth, we did not obtain data regarding the type of containers used for food storage or the type of cookware used for their preparation. Thus, it is possible that the MPs in the stool samples originated from the food itself or from cooking and distribution processes, given that human dietary patterns and cooking processes are highly variable. Seventh, the method of food packaging when eating out is unknown. Among the subjects in our study, only one person had eaten out, and that too only once. Therefore, the food packaging methods used when eating out are unlikely to have significantly affected the results of this study. However, future similar studies should take into account external food packaging. Therefore, more detailed studies involving a larger number of participants and taking into account food preparation and distribution processes are required to accurately identify MP exposure pathways.

## 5. Conclusions

In this study, we investigated the relationship between intake of food type, stool MP content, the levels of oxidative stress markers, and inflammatory cytokines. The results suggested a positive relationship between seafood intake and stool MP particle density. We also observed that stool MP content likely affected the level of TSLP, an inflammatory cytokine that is involved in colonic inflammation. Given the potential adverse effects of MPs on living organisms, it is desirable to reduce MP exposure. Particularly, based on the findings of this study, efforts aimed at reducing marine plastic pollution and the development of plastic-free distribution systems or those with reduced plastic packaging use are highly desirable.

## Abbreviations

AP1: activator protein 1
CI: confidence interval
DSS: dextran sulfate sodium
EAA: Ethylene-Acrylic Acid copolymer
EEA: Ethylene-Ethyl Acrylate copolymer
EVA: Ethylene-vinyl acetate copolymer
FT-IR: Fourier transform infrared spectrophotometer
GM-CSF: granulocyte-macrophage colony-stimulating factor
IFN: Interferon
IL: Interleukin
IO: Ionomer
LD-IR: Laser direct infrared spectroscopy
MPs: Microplastics
OR: Odds ratio
PE: Polyethylene
PEO: Polyethylene oxide
PET: Polyethylene terephthalate
PMMA: Poly methyl methacrylate
PMP: Polymethyl pentene
PP: Polypropylene
PS: Polystyrene
PU: Polyurethane
PVC: Polyvinyl chloride
PVS: Poly vinyl stearate
SI: Silicone
TSLP: Thymic stromal lymphopoietin
TD-GC-MS: Thermal desorption-gas chromatography-mass spectrometry with a tubular furnace

## Supplementary information

**Additional file 1:** **Supplementary Figure 1.** Distribution of number of total microplastic particles across tertiles of TSLP levels.

**Additional file 2:** **Supplementary Table 1.** Relationship between each stool microplastic particle density and seafood intake.

## Declarations

### Availability of data and materials

All data are incorporated into the article.
