# Supplementary material for: Relationship between microplastics in stool, diet, and inflammatory markers in healthy Japanese individuals
Source: Environ Health Prev Med. 2026 Mar 6;31:17. doi: 10.1265/ehpm.25-00403 (PMC12981973; doi:10.1265/ehpm.25-00403)
Supplement: Supplementary file 1 — Additional file 1: Supplementary Figure 1. Distribution of number of total microplastic particles across tertiles of TSLP levels. [file ehpm-31-017-s001.docx]

**Supplementary Figure1. Distribution of number of total microplastic particles across tertiles of TSLP levels.**
